# Supplementary material for: Effectiveness of FLASH vs. Conventional Dose Rate Radiotherapy in a Model of Orthotopic, Murine Breast Cancer
Source: Cancers (Basel). 2025 Mar 25;17(7):1095. doi: 10.3390/cancers17071095 (PMC11988084; doi:10.3390/cancers17071095)
Supplement: Supplementary file 1 [file cancers-17-01095-s001.zip › cancers-3487754-supplementary.pdf]

Supplementary Information

Supplementary Table S1. *p*-values from tumor volume comparisons between FLASH and CONV irradiation groups. *p*-values were derived from Mann–Whitney U tests performed at each indicated day post-irradiation, comparing tumor volumes between FLASH and CONV groups. Analyses are presented separately for Round 1 (20 Gy and 30 Gy), Round 2 (20, 25, and 30 Gy), and merged datasets from both rounds. Unless otherwise specified, all analyses correspond to small size tumors (20–40mm^3). The 30 Gy (Lg) group represents the analysis of large size tumors (250-800 mm^3). Statistical significance was set at α = 0.05.

DAY POST RT	ROUND 1 20Gy	ROUND 1 30Gy	ROUND 1 30Gy (Lg)	ROUND 2 20Gy	ROUND 2 25Gy	ROUND 2 30Gy	MERGED 20Gy	MERGED 30Gy
**0**	0.885714	0.342857	0.818182	0.142857	0.804662	>0.999999	0.243978	0.243978
**2**	0.485714	0.771429	0.937229	0.714286	0.005905	0.685714	0.48345	0.48345
**4**	0.485714	>0.999999	0.937229	0.914286	0.895105	0.114286	0.409324	0.409324
**6**	0.971429	0.142857	0.818182	0.571429	0.82906	0.371429	0.773893	0.773893
**10**	0.657143	0.828571	0.484848	0.371429	0.577156	0.142857	0.253768	0.253768
**12**	0.257143	>0.999999	0.484848	>0.999999	0.82906	>0.999999	0.307071	0.307071
**16**	>0.999999	>0.999999	0.818182	0.314286	0.140637	>0.999999	0.706294	0.706294
**23**	>0.999999	>0.999999	0.588745	0.342857	0.043823	>0.999999	0.647552	0.647552
**30**	>0.999999	>0.999999		0.2	0.271062	>0.999999	0.386325	0.386325
**33**	>0.999999	>0.999999		0.7	0.062771	0.428571	0.5338	0.5338
**39**	0.914286			0.4	0.309524	0.828571	0.729021	0.729021
**42**	>0.999999			0.7	0.32684	0.828571	0.83683	0.83683
**46**	0.914286			0.8	0.132035	0.828571	0.848485	0.848485

Supplementary Table S2. Mean rank differences between FLASH and CONV groups at each post-irradiation day. Mean rank differences (FLASH minus CONV) were calculated from Mann–Whitney U tests at each indicated time point post-irradiation, with positive values indicating higher tumor volumes (higher ranks) in the FLASH group and negative values indicating higher tumor volumes in the CONV group. Unless otherwise specified, all analyses correspond to small size tumors (20–40mm³). The 30 Gy (Lg) group represents the analysis of large size tumors (250-800 mm³).

DAY POST RT	ROUND 1 20Gy	ROUND 1 30Gy	ROUND 1 30Gy (Lg)	ROUND 2 20Gy	ROUND 2 25Gy	ROUND 2 30Gy	MERGED 20Gy	MERGED 30Gy
**0**	0.5	2	-0.6667	-2.625	0.6696	0	-2.813	-2.813
**2**	-1.5	0.75	-0.3333	-0.875	-6.161	-1	-1.741	-1.741
**4**	1.5	0	0.3333	0.5833	0.4018	-3	2.009	2.009
**6**	-0.25	-3	-0.6667	1.167	0.5357	1.75	0.8036	0.8036
**10**	1	0.5	-1.667	1.458	1.339	2.75	2.679	2.679
**12**	2.25	-0.25	-1.667	0	-0.5357	0.5	2.411	2.411
**16**	1	0	-0.6667	-2.042	3.214	1	-0.8036	-0.8036
**23**	1	0	-1.333	-1.75	4.554	1	-1.071	-1.071
**30**	0.5	0		-2.333	2.625	1	-2.009	-2.009
**33**	0	0		-1	4	2	-1.429	-1.429
**39**	0.5			-1.667	2.333	0.5833	-0.8571	-0.8571
**42**	0			-1	2.167	0.5833	-0.5714	-0.5714
**46**	0.5			-0.8333	3.333	0.5833	0.4643	0.4643

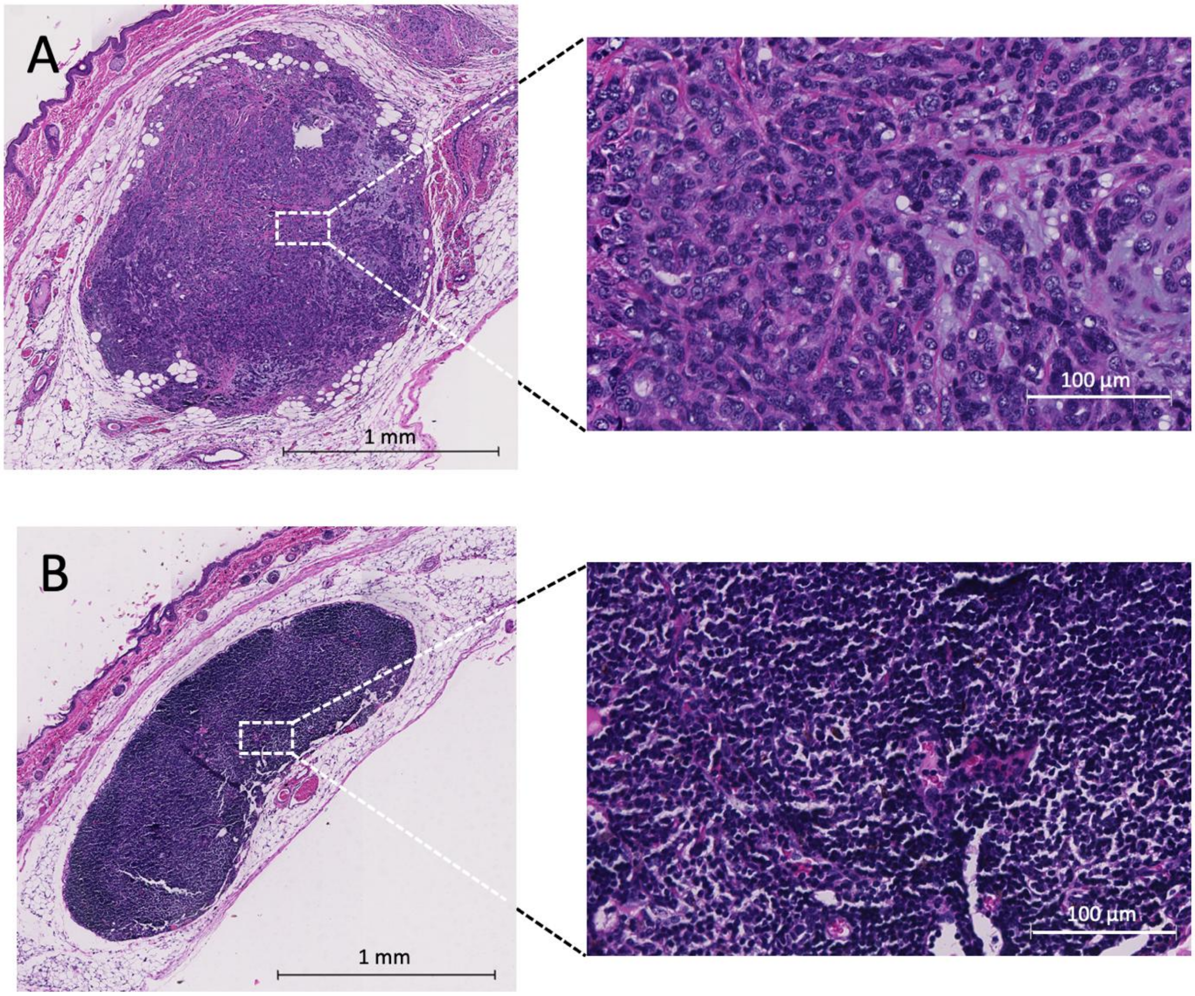

Supplementary Figure S1. Characterization of mammary gland tissue and lymph node post-exposure to irradiation at day 46. (**A**) Histological analysis of a skin stripe from the fourth mammary fat pad, exhibiting a Py117 breast cancer orthotopic tumor. The presented photomicrograph, stained with Hematoxylin and Eosin, displays the tumor, which has a caliper-measured volume of 41.83 mm^3. Blow up image highlights the cellular phenotype within the tumor. (**B**) Histological view of a lymph node situated in the implantation region of the Py117 breast cancer orthotopic tumor. Despite being accounted for as part of the tumor volume, with a caliper-measured volume of 24.63 mm^3, the lymph node did not exhibit the presence of breast cancer cells upon histopathological evaluation. Blow up image highlights the cellular phenotype of the lymph node. At very small tumor volume measurements with calipers, an inflamed lymph node can be a source of false positive for residual tumor and contribute to the noise in small tumor volume measurements.
